# Irritable Heart

**Published:** 1920-06

**Authors:** C. F. Coombs, C. E. K. Herapath, A. G. Morris


### IRRITABLE HEART.

At a discussion at the Bristol Medico-Chirurgical Society on March 10th, 1920, the subject of "irritable heart" was examined by different speakers from different points of view.

Opening the discussion with a short survey of the condition as seen in civil practice, Dr. C. F. Coombs said:—

It was during the latter part of 1918 that I became aware of an increase in the incidence of the irritable heart syndrome among the civil population.

The usual complaint is of uncomfortable sensations arising in the præcordial area. These are variously described, but the general term "palpitation" fairly includes them all. Other symptoms are shortness of breath, pain in the left chest and arm, and faintness sometimes amounting to syncope.

The palpitations in bad cases are practically continuous, but more often they are limited to attacks which are brought on by emotion or exertion, with undue readiness in the patient's own judgment. Sometimes they seem to be related to meals.

One is often told that the attacks come on when the patient is lying in bed at night. They cause less dyspnœa than one would expect, but sometimes they are accompanied by an irregular, sighing kind of breathing. If the patient has an organic lesion of the heart in addition to his bouts of palpitations, he is, of course, liable to shortness of breath in the attacks. Pain is not often severe. The patients who put pain most definitely in the foreground of their story are elderly. The faints are of an interest to be alluded to presently.

The most constant objective feature is tachycardia. This is the source of the præcordial discomfort of which the patient complains, and like it is continuous in bad cases. More often it is occasional, excited with undue ease by emotion, especially of the apprehensive type, by movement from a lying to a sitting or standing attitude, and by exertion. I give these stimuli in the order of their importance as it has appeared to me. Under these influences the pulse shoots up from 90 to 120 or even 140, and runs away at that rate even after the stimulus has been removed. In some cases periods of retardation may intrude more or less suddenly into the tachycardia, accompanied occasionally by extra-systoles. It is in these patients that faints occur. In a case seen recently the pulse was observed to become slow during a series of fainting attacks. Sometimes retardation is a more striking feature than acceleration. A high systolic pressure is occasionally noted, but even in such cases the diastolic pressure is normal or low. In 90 per cent. of the cases I have analysed for this paper there were no signs whatever of structural disease of the heart; in those with physical signs the lesion was trivial in all but one, and in none of them was there reason to regard it as the cause of the quick pulse. The arrhythmia set up by the see-saw between acceleration and retardation proves on graphic analysis to be a disturbance of the whole heart, *i.e.* it is impressed on the heart through its nervous control and not by faults within the heart itself. The favourable course of the cases also supports the view that the syndrome has nothing to do with structural disease of the heart.

To what then is "irritable heart" due? In order to answer this question, if possible, I have analysed 50 cases seen consecutively and at such leisure as to allow of careful inquiry. 13 of these 50 patients, though seen in civil practice, had acquired their troubles as a result of military service. 9 of these were men, 4 women; their average age was 27. But considering all the 50 cases together, I find the sex incidence about equal and the average age 38. Three-fourths of the cases fell into the 3rd, 4th and 5th decades. Puberty might be suspected as causal in 4, and the menopause in a like number. Only half a dozen of the 50 were occupied in heavy manual labour.

Among my hospital patients the wives and widows of soldiers bulk largely. Physique is less important than I should have anticipated; the irritable heart of adolescence is so often associated with a tall, lanky build that I had expected to find some similar relation to be general. As a fact, only 9 of the 50 were people of particularly slim physique.

There is, however, a strong temperamental predisposition ; about half the people were, either by inheritance or personally or both, of the "high-strung," introspective cast of mind.

As to exciting causes, I inquired particularly into the incidence of infection and of stress, physical and emotional. In 32 cases there was a history of infections that bore some conceivable relation to the onset of symptoms. I have included those with a remote history of rheumatic infection in this group because much has been made of a latent myocardial weakness following rheumatic infection as a factor in the production of irritable heart. But I do not believe much in this idea. The myocardial lesions of rheumatism are relatively short-lived except in severe cases, and my series of 50 includes no severe cases of post-rheumatic heart disease. In 22 cases the time relation between the infection and the onset of symptoms was close enough to make one believe in the causality of the former, and in 14 of these the particular infection seems to have been influenza or pneumonia.

As to physical stress, not even the most generous definition of the term could find for it more than 11 examples.

Emotional strain was apparent in the histories of 36 of the patients. This is probably a low estimate, as it only includes those cases in which the facts were undeniable. The sources of emotional unrest were about equally divided between anxieties regarding the fate of relatives on war service, anxieties caused by serious illness at home, domestic discord, business worries and litigation, prolonged war service in dangerous areas, and the concealment of facts of which the patient was ashamed. All these appear to me to be varieties of protracted fear—fear of something anticipated rather than of something actually present.

Macbeth speaks of—

> "That suggestion
> Whose horrid image doth unfix my hair
> And make my seated heart knock at my ribs,
> Against the use of nature. Present fears
> Are less than horrible imaginings."

It seems, then, that two factors are of prime importance in the establishment of this syndrome, infection and emotion, both of them, be it remarked, forms of reaction on the part of the patient to unfavourable environment. My impression is that either factor may make the heart irritable without the other, but that the worst cases are those in which both are acting together.

That it is upon the nervous control of the heart that they act rather than on the heart itself is generally agreed, but as to the precise point of the controlling apparatus on which they act there is no such consensus. Indeed, books have already been written on the matter. The question is, however, no easy one. The answer can only be furnished by prolonged research. Such an investigation would be of considerable value not only for its own sake but because of the light it might throw on cognate problems.

Whatever may be the part of the nervous system upon which these injurious influences impinge, there is no doubt as to the line of treatment to be adopted, and that is to attack the cause. Any infection still persisting must be identified and treated either by direct means or by enhancing the patient's capacity for resistance. Sources of painful emotion have to be looked for, and the patient must be told that these are largely responsible for the symptoms, so that he may be induced either to put right the situation which is disturbing his mind, or if this is impossible to adapt himself with equanimity to his conditions.

Finally, the attitude of the patient to his symptoms varies widely. Many persons have a tachycardia exactly like that of these patients without distressing themselves about it. Sometimes they understand its significance and are therefore not frightened by it; often other symptoms arising from the same source, whether physical or infective, dominate the mind and the sense of cardiac discomfort is subordinated to these. But in many the cardiac sensations excite mental disturbance amounting sometimes to a terror that serves to aggravate the tachycardia. In order to do good to such patients it is essential to explain to them the nature of the disturbance, laying stress on its harmlessness, and asking for active co-operation in a campaign against its causes, whether emotional or infective.

Dr. C. E. K. HERAPATH next treated of the same syndrome as seen among ex-service patients. He said:—

For the last two months I have seen all cases referred from Medical Referees and Pension Boards with symptoms of heart disease, and these notes are the outcome of the first 57 cases.

Of these 57 men 9 had enlarged hearts, 5 of them with murmurs indicative of aortic regurgitation, 3 of mitral stenosis and 1 of both conditions. The remaining 48 presented symptoms of irritable heart, and it is of these that I propose to speak to-night.

The history of the onset and the causation thereof was carefully gone into, and it was found that 28 cases began within two weeks of being allowed up as convalescents from definite illnesses, influenza in 7 cases, trench fever in 5, malaria in 4, rheumatic fever in 3, pneumonia, typhoid group, bronchitis and gassing in 2 each, and dysentery in 1. Four cases followed wounds and accidents. The remaining 16 patients had neither illnesses nor accidents. Of these 11 gave a definite history of losing their nerve some time before the onset of symptoms, 3 had a history of shortness of breath before enlistment, and in 2 no cause could be found. Of the whole 48 men 27 were doing heavy work before the war, whereas 21 were in light or sedentary occupations.

In a typical case the man complains that the least exertion brings on shortness of breath, palpitation, a stabbing or shooting pain over the præcordium or sternum, occasional attacks of giddiness or actual fainting, and sweating, particularly in the hands and axillæ. He suffers from nervous symptoms, such as tremor, loss of sleep, dreaming, excitability, irritability of temper, and emotionalism. Many of them cannot go to picture theatres, as the excitement brings on palpitation and pain. The pulse-rate is 100–120 or more.

The examination is carried out with the man first standing, then he lies down on a couch. This brings the pulse-rate down about 8 beats a minute. While he is lying his blood-pressure is taken. He then gets up and does exercise (double marking time), until he feels he must stop. The pulse-rate goes up to 130–180. Then he lies down again, and the pulse is found to drop to 90–100. It is interesting to note that practically always the rate falls below what it was before exercise. Frequently it is suddenly halved, and in a certain number of cases premature contractions occur during the slow period. The examination usually shows the heart to be smaller than normal, various murmurs may be detected, and the systolic blood-pressure is high.

This is the average case with which one meets, and I shall now enlarge upon a few of these phenomena.

With reference to the size of the heart, I take the position of the maximum thrust of the apex against the chest wall as the best guide. If this be inside the mid-clavicular line there is no enlargement of the ventricles, provided the heart is not displaced as a whole. If the apex-beat be not palpable I give the patient a little exercise to strengthen the beats, and in most cases this is effectual. I do not think percussion is a reliable index where the apex-beat cannot be felt, as lung is probably overlapping the left border of the heart, and impairment of percussion note will then not give the position of the left margin.

In these 48 cases there were 9 of enlargement of the heart, 4 with general arterial sclerosis, 2 with chronic interstitial nephritis ; 2 of them had complained of shortness of breath from childhood, and probably had an unsound myocardium when they enlisted. In one case no cause of enlargement could be found.

These cases should be looked upon as cases of " irritable heart " complicating other disease, for a large number of the patients with no enlargement have had symptoms for three or four years and 2 cases for five years ; whereas 3 of the men with enlarged hearts have had symptoms for less than eighteen months and 3 for less than two years, so that it does not seem that continuance of tachycardia causes enlargement.

The average position of the apex-beat is 3 inches from the middle line, the normal being from $3\frac{1}{2}$ to $4\frac{1}{2}$ according to the development of the chest, or rather of the man. In other words, the heart is smaller than normal ; and this is what one would expect, for a rapidly-beating heart does not have time to fill properly, and therefore does not fully relax, consequently the output per beat is small.

A diffuse apex-beat is very frequently met with ; in fact, movement of the chest wall can be seen over a large area of the præcordium. A diffuse apex-beat does not mean dilatation, although a dilated and therefore failing heart may have a diffuse apex-beat.

On auscultation a short, sharp, loud first sound is heard and usually a high-pitched second sound, though if the heart is beating very rapidly the second sound is not easily heard at the apex. A variety of murmurs may be present ; a systolic at the apex or on either side of the sternum at the base is very common. Some occur only during inspiration, a few only during expiration. Many which are heard during both are not heard when the breath is held. Some appear in a standing position, others on lying down, some only appear when the heart is beating rapidly. Others are sometimes present and sometimes absent in the same patient over quite short intervals of time. In one case a diastolic murmur was heard over the third left costal cartilage only during expiration. Some of these murmurs are cardio-respiratory, some are hæmic, at a high pulse-rate a relative incompetency may occur, probably partly mechanical and partly protective. A murmur which never varies may be due to a lesion of a valve, or to a relative incompetency from dilation of the mitral ring, or to other causes. It is impossible in many cases to say positively that a systolic murmur is or is not due to a damaged valve. The prognosis is not affected by our ignorance so long as there is no evidence of myocardial disease.

The systolic blood-pressure is moderately high; 48 mm. is the average of cases without enlargement. The diastolic pressure is low, giving an abnormally high pulse-pressure, usually 50–70 mm., though I have met with one case where the pulse-pressure was 100 mm. The low diastolic pressure is due, I think, to dilatation of the arterioles, inasmuch as with the higher pulse-pressures capillary pulsation and the water-hammer type of wave is met with in cases where aortic regurgitation is out of the question.

Then as to the rate. No doubt this goes up from nervousness when the patient comes into the room. But although I make a point of taking the history, getting them to talk and otherwise distracting their attention, the rate does not come down appreciably. Nor when they lie down does the tachycardia tend to quieten more than a few beats per minute, but almost invariably after exercise and lying down the rate is the slowest since their entry into the room. On listening to the heart when the patient lies down one can appreciate the fact that the vagus is " putting the brake on," and this occurs during successive expirations. Thus the first slowing is noticed during expiration. The rate quickens during the following inspiration, but slows still further during the next expiration, and so on till the rate comes down to 90 or 100. After a longer or shorter period of slowing the rate gradually goes up again.

It would seem that while the man is up and about the vagus action is prevented, but that exercise followed immediately by rest awakes or releases the activity of the vagus centre. The same thing occurs if the patient remains standing, but not nearly to the same extent, and it takes much longer. If exercise thus ameliorates the condition it is obvious that the heart muscle is good.

I have taken several polygraph tracings of the premature contractions that occur during the slow period, and find that both auricular and ventricular premature beats may occur. These are, perhaps, due to overfilling of the ventricles during the lengthened diastole, though the increased vagal action may have something to do with it. Sudden halving of the rate, which frequently occurs, is due to the vagal action cutting out alternate stimuli at the sino-auricular node. Electro-cardiograms have always shown that the tachycardia is composed of normal beats ; that is, of orderly contractions of the whole heart initiated from the sino-auricular node.

To sum up briefly. In 39 out of 57 cases in this series we were dealing with rapidly-beating hearts which were not enlarged, and which were contracting in normal sequence from the sino-auricular node downwards. In other words, the fault does not lie in the heart itself, any more than the tachycardia of Graves's disease does. Where then does it lie? Possibly in the nervous system. In 50 per cent. of cases one finds that with the onset of the shortness of breath the nervous system becomes upset. Sleep is disturbed, dreams of a horrible and frightening nature occur, the patient becomes irritable, loses his nerve, funks going back into the line and becomes very emotional. Unfortunately, owing to army nomenclature his case becomes diagnosed "D.A.H.," and from that day onwards believes he has heart disease. This worries him horribly, and he comes to live in a vicious circle. His nervous disturbance increases the tachycardia and the tachycardia depresses him, makes him anxious and increases the nervous strain and so on. Finally he becomes hopelessly neurasthenical or psychasthenical.

The problem in treatment is to break the vicious circle. First, after a careful examination the man must be assured that the heart is sound, that it beats quickly on exertion because the nerves leading to the heart are irritable and cause the heart to beat too quickly. Then one should point out how exercise slowed the heart and get him to devote half an hour each day to physical exercises, alternately performing an exercise till he gets short of breath and then resting and so on. He must be told that if he does this systematically he will be able to do more and more before he gets short of breath. I have seen enough improvement take place on this system during the short time I have been at the work to make me distinctly hopeful of effecting a cure in the majority of cases. Naturally any infection such as bad teeth, a suppurating ear, malarial attacks or chronic dysentery should be cleared up, as toxins absorbed into the system seem to play a large part in bringing on the syndrome, and may therefore be the means of prolonging it. Drugs do not as a rule do much good, though in the higher irritable cases bromides are useful in moderate doses.

Dr. A. G. Morris contributed notes on four soldiers observed at Southmead. A., B., and C. were all men who had had no service overseas. Previous to joining the army two were working in sedentary occupations as draper's assistants, the third was a different type—a farm labourer. All three were working in or near London, and on joining the army were stationed in Kent for training purposes. Their previous history showed no trace of any illness, there was no record of any source of infection either before or after they joined the army, they had been able to play football and other games, and were passed "A" into the service.

During the period of their training there was no disability, and they were able to carry out their duties with regard to full infantry training without any discomfort. For some reason, certainly not on health grounds, probably general usefulness, they were retained at their original training depot for a longer period than was usual, but at last they were transferred to a depot farther away, from which drafts were being continually sent overseas

Now this interesting thing happened, that in spite of their training in this depot being much lighter than previously it was from this period that their disability "D.A.H." and its syndrome definitely dated, and it was from this depot that they were sent into hospital and reduced in category. Up to this time they had been stationed near their homes, and had been carrying on with their full training and military duties without any physical inconvenience. Shortly after joining this latter depot each one gave the same history of palpitation, headache, dyspnœa and præcordial pain on emotion, tachycardia and collapse on the march. Here we have the mental factor at work, namely while they had been undergoing full training at one of the biggest training camps with the expectation of being drafted overseas, this prolonged detention in England so near their own homes, which they were able to visit frequently, set up a curious psychic effect, caused by the pull of on the one hand home and family, on the other hand army life and service overseas.

This state of affairs was suddenly brought to a climax by their transference to a depot from which they were daily expecting to be sent overseas. For the remainder of their time before admission to Southmead they were employed on light duty with occasional visits to hospital.

On admission to Southmead they all showed practically the same symptoms and signs: præcordial pain, palpitation and dyspnœa on exertion, no enlargement of the heart, sounds clear and regular, no murmurs, but marked and increasing tachycardia, pulse return good. In one case conscious exertion produced pallor and extreme dyspnœic distress. Here again the strong psychic element was plainly illustrated. This particular man was told to walk twice round the hospital at a good pace, his pulse having been taken with a certain amount of ostentation before starting. He was observed through the ward windows carrying this out most conscientiously with a look of resigned misery on his face, and on arrival he was in a state of marked dyspnœa and tachycardia, and pulse about 150. This same man on another occasion, after his pulse had been taken at the usual daily time by the nurse, was told to hurry to the public-house about a quarter of a mile away to get two bottles of beer for the ward. He executed this quite quickly, and arrived back with scarcely any dyspnœa, his pulse being taken the moment he arrived in the ward before he realised what was happening, and the rate was about 90.

The fourth man, D., was an R.A.M.C. orderly stationed at a home hospital. Previously he was a clerk in the same town. He had always been healthy, in fact from time to time had done fairly heavy work in the factory. Here again we have a similar history. For eighteen months after joining the hospital he carried out the full duties of an R.A.M.C. orderly, lifting patients and carrying stretchers without any marked physical inconvenience. Suddenly all the personnel were boarded with a view to their fitness for transference to infantry or overseas drafts. He was boarded, told "there was something wrong with his heart," and marked B 1 (obviously it was not a gross lesion). From this time his disability dates. As the three previous cases, his symptoms were palpitation, dyspnœa, pain over the heart. In spite of treatment from time to time, including one period of two months at a seaside convalescent hospital, which on his own showing made him worse, he developed more and more symptoms. In the words of a Sister at his hospital, "D. was one of the best orderlies I have had during the war until he had a medical board, after that he was utterly useless, afraid to move hand or foot and full of complaints." On admission to Southmead he had the typical anxious expression, enumeration of symptoms *ad infinitum*, extreme tachycardia on being examined, and he complained of breathlessness, palpitation and pain over the heart. The first week he was in the hospital his pulse was taken by the nurse night and morning, and never exceeded 80 standing; taken by the M.O. about 120, rising rapidly. The heart showed no enlargement, sounds clear, no murmurs, occasional extra-systoles confirmed by polygraph, exercise tolerance fairly good but extreme dyspnœa. On conscious exertion, fine tremor of hands, reflexes brisk, general mentality excitable.

Cases of a similar nature were observed at a base in France over a period of eighteen months. On reviewing the board sheets at the A.I.D.'s[1] periodic reclassification boards, it was noticed that in a large number of cases the original disability retaining men at the base as "P.B." or "P.U." was of such a kind as deformed or flat feet, hernia, poor physique,

[1] A.I.D.=Assistant Inspector of Drafts.

etc. Gradually there crept in the well-known symptoms and signs in addition to the original disability, and the diagnosis became "flat feet and D.A.H." In some cases the first disability dropped out of the diagnosis as the "D.A.H." syndrome became more pronounced, and "D.A.H." alone remained as the diagnosis. A further stage even was reached in a few cases when the symbols "D.A.H." were converted into "V.D.H." The etiology of this is not easy to define; probably the comforts, not to say luxuries of the base, weighed against the natural, almost unconscious ever-present dread of the line.

Some sports were held at a certain hospital during the above-mentioned period. The winner of the 100 yards race in fairly reasonable time was found to be classified "B3" for hernia and "D.A.H."

No reflection is intended on either the M.O.'s who boarded or the men who suffered under these all too real complaints, but there is no doubt that a very strong psychic element was present. Unfortunately, we were too ready with the diagnosis of "D.A.H," and this caused untold misery to numbers of men who found in it a confirmation of their fears, long engendered by their constantly recurring cardiac symptoms. It was by this means that so many men, to all intents and purposes healthy, have become physical wrecks, afraid to work, afraid to play, a misery to themselves and others, as is well seen on the present-day Pensions Boards.

Dr. J. A. Birrell read notes on four cases of **Post-influenzal tachycardia** now at the General Hospital.

1. The first patient, A. B., married woman, aged 40. Ten months ago had an influenzal attack, which was preceded by anxiety about the possibility of her husband being called up for the army, and the worry of getting food for her family. Since the illness this had developed into morbid apprehension and anxiety; physical effort caused a sense of exhaustion and there was persistent sleeplessness. *On examination* there was general tremor, especially of the tongue, nervousness, and a marked tendency to flushing. The pulse was at first 96, but quickened remarkably during the interview, sometimes going up to 160, the woman becoming restless and turning about in bed, obviously ill at ease. However, when taken by the Sister in the ward, the rate was not usually above 100. The heart-rate was not slowed by swallowing, vagal compressions, or digitalis, nor was it accelerated by atropine. There were no physical signs of disease.

2. Showing a condition similar to the above was C. D., a married woman, aged 36. Twelve months previously she had influenza, during a period of anxiety, like A. B., about her husband being called up. Since then, in her own words, she had "worried over the smallest thing." She was conscious of cardiac quickening on the approach of her doctor, and she was unable to sleep well. Rest in bed for ten days six months ago did not bring about any improvement. *On examination* she was obviously nervous, and in a state of general tremor. The pulse-rate was 144, and the systolic blood-pressure 110. There were no signs of cardiac disease.

3. The other subjects were men. E. F., a riveter's assistant, aged 41, had influenza thirteen months before, after a period of overwork and financial worry. Since then he had been nervous, sleepless, and apprehensive and, as he says, "unable to sit down quietly to a meal." He is conscious of pulse quickening on excitement. *On examination* he was not obviously nervous or agitated, but there was slight tremor of the hands. The pulse was 100, and was not much quickened by exertion. His heart appeared free from disease.

4. G. H., aged 39, dock labourer, was overworked and worried about his wife, who was ill at home, and whom he had to nurse twelve months before examination. Then he was himself attacked by influenza. Since then, he says, "his nerves had been bad," and he had been "shaky on excitement," and he knew that hurrying would cause, as he termed it, "jumpiness," and cardiac fluttering. He had been unable to sleep well, and felt easily exhausted. *On examination* he was collected and steady in manner, but the hands and tongue were tremulous. The pulse varied in rate from 120 to 150 when at rest, rising to 200 on exertion, but there was no accompanying dyspnœa or distress, and his heart showed no signs of disease.

These four patients showed tachycardia, preceded about twelve months ago by influenza. In three instances the statement was volunteered that the illness occurred during a period of mental distress and anxiety. In the fourth this fact was readily elicited in conversation.

Prominent amongst the symptoms were nervous apprehension about small matters of everyday occurrence, sleeplessness, a feeling of exhaustion and despair, and a sensation of cardiac acceleration interpreted as a fluttering in the chest, a voluntary statement often being given about this condition on taking the pulse when first examining the patient.

Objectively these people were tremulous in the tongue and hands. The pulse was quick but regular. It was not found possible to slow the heart by vagal compression or swallowing, or by digitalis in maximal doses. So quick was it even when the subject was at rest that there was usually no marked acceleration on exertion or on the administration of atropine. All four patients were at work up to the time of examination, in the case of the two men hard manual labour. There were no abnormal physical signs detected in the heart itself, and polygrams showed that the quickening involved the whole heart.

